# The Odd Couple(s): An Overview of Beta-Lactam Antibiotics Bearing More Than One Pharmacophoric Group

**DOI:** 10.3390/ijms22020617

**Published:** 2021-01-09

**Authors:** Margherita De Rosa, Anna Verdino, Annunziata Soriente, Anna Marabotti

**Affiliations:** Department of Chemistry and Biology “A. Zambelli”, University of Salerno, 84084 Fisciano (SA), Italy; averdino@unisa.it (A.V.); titti@unisa.it (A.S.)

**Keywords:** bis-azetidinone derivatives, dual antibiotics, podands, siderophores, bis beta-lactam, bis beta-lactam macrocycles

## Abstract

β-lactam antibiotics are among the most important and widely used antimicrobials worldwide and are comprised of a large family of compounds, obtained by chemical modifications of the common scaffolds. Usually these modifications include the addition of active groups, but less frequently, molecules were synthesized in which either two β-lactam rings were joined to create a single bifunctional compound, or the azetidinone ring was joined to another antibiotic scaffold or another molecule with a different activity, in order to create a molecule bearing two different pharmacophoric functions. In this review, we report some examples of these derivatives, highlighting their biological properties and discussing how this strategy can lead to the development of innovative antibiotics that can represent either novel weapons against the rampant increase of antimicrobial resistance, or molecules with a broader spectrum of action.

## 1. Introduction

**Figure 1:**
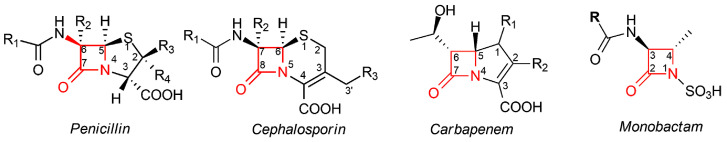
Core structures of different classes of β-lactam antibiotics. In penicillins, the β-lactam moiety is fused to a five-member thiazolidine ring; in cephalosporins, the β-lactam moiety is fused to a six-member dihydrothiazine ring; in carbapenems, the β-lactam moiety is fused to a pyrroline ring; in monobactams, the β-lactam ring is not fused to any other ring. In all classes, the β-lactam moiety is highlighted in red; the standard numbering of the nucleus for each class is also reported.

β-lactam antibiotics are a broad group of molecules that are naturally produced by different organisms (molds belonging to *Penicillium* spp. and *Cephalosporium* spp. for penicillins and cephalosporins, respectively, and bacteria belonging to different species for monobactams and carbapenems) (Figure 1).

The serendipitous discovery of the first representative of this broad group of molecules, penicillin G, is generally attributed to Alexander Fleming in 1929 [1], although there are pieces of evidence that, about fifteen years before, an Italian physician, Vincenzo Tiberio, had already identified in molds a “principle with bactericidal action” [2]. Only in 1949, however, was the structure of this compound fully revealed, thanks to the pioneering X-ray studies of Dorothy Crowfoot Hodgkin [3]. Another Italian pharmacologist, Giuseppe Brotzu, identified the presence of other compounds with bactericidal activity in crude filtrates of *Cephalosporium* cultures in 1945 [4]. The founder of this second class of compounds, cephalosporin C, was identified from these filtrates in 1953 and its structure was fully characterized in 1961 [5,6]. The other classes of β-lactam antibiotics, namely carbapenems and monobactams, were discovered more recently [7,8], in an effort to identify new members of this broad group of molecules. 

All these compounds share a common chemical moiety, i.e., a four-member ring with an amidic function, commonly called “β-lactam ring” or “azetidinone”. In penicillins, cephalosporins, and carbapenems, this ring is fused to another 5- or 6-member ring, whereas in monobactams, the β-lactam ring is monocyclic (Figure 1). This moiety is the mainly responsible for the antibacterial properties of all these molecules, due to their ability to block the bacterial cell wall synthesis as a result of their covalent binding to penicillin-binding proteins (PBPs), which are essential enzymes involved in the terminal steps of the synthesis of peptidoglycan, the main component of the bacterial cell wall [9].

After the so-called “Golden Age” of antibiotic discovery (from the 1940s to the 1960s) [10], researchers had to realize the progressive increase of microorganisms that are not susceptive to the action of these drugs. This resistance is caused by the development of many different “escape strategies”, such as the alteration of the target, the development of pumps to export the antibiotics outside the microbial cell, and in particular for β-lactam antibiotics, the widespread diffusion of β-lactamases, enzymes able to inactivate their pharmacophoric core by hydrolysis of the β-lactam ring [11]. Therefore, during the last decades, a huge effort has been made worldwide to identify or synthesize new antibiotics, able to encompass the antimicrobial resistance that has been recently declared by WHO “one of the top 10 global public health threats facing humanity” (https://www.who.int/news-room/fact-sheets/detail/antimicrobial-resistance; declaration of 13 October 2020). Additionally, several derivatives of the natural compounds have been created to modulate their pharmacokinetic properties and/or to widen their spectrum of action. For example, to date, hundreds of cephalosporin derivatives are available and they are classified into five “generations” that are characterized by different ways of administration and by an enhanced microbicidal activity towards Gram-negative bacteria (while the founder molecule is mainly active on Gram-positive bacteria) [12]. 

The total synthesis of β-lactam antibiotics in the laboratory is difficult because these molecules, despite their limited size, are often extremely complex in function and chirality [13]. Therefore, the vast majority of the chemical modifications applied to β-lactam antibiotics involve the functionalization of the pharmacophoric core, typical of each class, using the total chemical or enzyme-assisted strategies. Each of the 1, 2, 3, or 4 positions on the β-lactam ring (Figure 1) have been considered for the introduction of numerous and different substituent groups in order to modulate the possibility of interactions with their targets, and consequently, to improve the biological activity.

In addition to the functionalization of the pharmacophoric core of β-lactam antibiotics, the development of bi- or multifunctional antibiotics is an interesting strategy to both increase the bactericidal activity of these compounds and to enhance their resistance to β-lactamases. In this review, we would like to summarize the efforts made during the years to create two kinds of bifunctional derivatives: those in which two or more azetidinone rings have been associated in a single molecule, and those in which the β-lactam moiety has been associated to other pharmacophoric moieties with different modes of action. We will describe some of the most representative molecules obtained so far. We would like to point out that, in this review, we will not include those pro-drugs in which a new function has been inserted to merely improve pharmacokinetics of original β-lactam compounds.

## 2. β-Lactam Antibiotics Bearing More Than One β-Lactam Ring

An interesting approach aimed at improving the biological activity of β-lactam antibiotics and fight resistance against them has been the introduction of more than one β-lactam unit inside the same chemical structure. The rationale behind proposing this strategy was to consider a possible synergistic action between the various β-lactam rings, which are capable of improving antibacterial activity and stability. 

**Figure 2:**
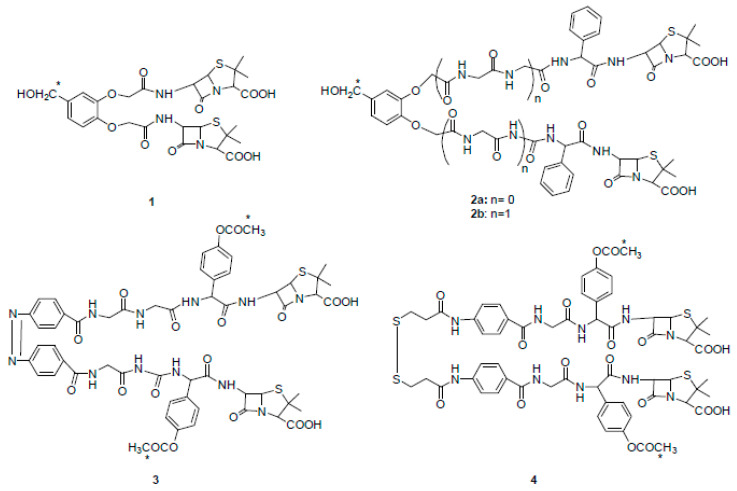
Chemical structures of radio-labeled bis-β-lactam compounds **1**–**4** (ref. [14]). Compound **3** is drawn in cis configuration owing to space limitations, although its configuration is trans. * indicates the radio-labeled atoms.

In the early 1980s, Rodríguez-Tébar and coworkers reported the preparation of different radio-labeled bis-β-lactams and a study on their interaction with *Escherichia coli* PBPs [14]. They synthesized bis-functional compounds derived from common β-lactam antibiotics such as 6-aminopenicillanic acid (6-APA), ampicillin, and amoxicillin. All derivatives (compounds **1**–**4**) showed PBPs binding affinities that were higher than those of their parent precursors and some of them were able to cross-link two molecules of PBPs (Figure 2).

**Figure 3:**
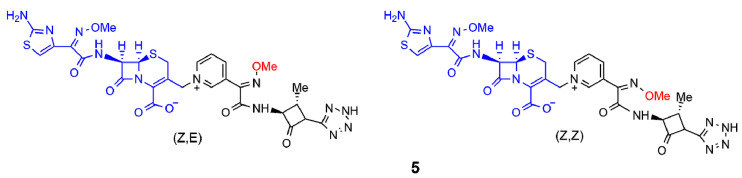
Selected β-lactam antibiotics synthesized by Panunzio et al. (ref. [15]). The moiety highlighted in blue is the portion derived from cefotaxime, the one in black is the aztreonam derivative. The methoxy-imine group mentioned in the text is reported in red.

Next, Panunzio and coworkers reported the synthesis of a series of novel compounds derived from the coupling between cefotaxime and different monobactams [15]. The structure of the monobactams was planned from the structure of aztreonam, a commercially available antibiotic drug, and replacing, at the N-1 position, the sulfonic acid group with a neutral tetrazole ring, and a pyridyl group at the C-3 position to obtain compounds similar to the third generation of antibacterial agents. Only compound **5** is shown in Figure 3 and it exhibited activity against most bacterial strains compared to the standard drug cefotaxime. In particular, the stereoisomer (Z,Z) of compound **5** was more active than both the reference drug and (Z,E)-**5** against one strain of *E. coli* and one strain of *K. pneumoniae*. This result was in agreement with the hypothesis of the synergic action of the two moieties. Indeed, (Z,E)-**5** and (Z,Z)-**5** differed only in the geometry of the double bond of the methoxy-imine in the monobactam moiety. As a consequence, when the monobactam from (Z,E)-**5** was released, it was inactive, while in the case of (Z,Z)-**5**, it was active.

**Figure 4:**
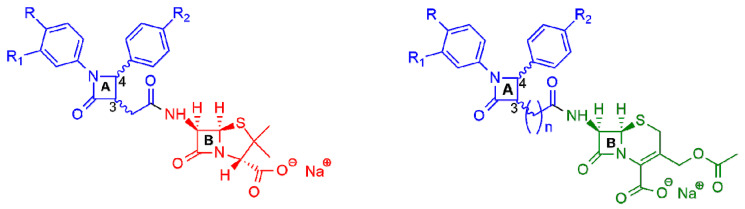
General structures of new semisynthetic penicillin and cephalosporin derivatives synthesized in Refs. [16,17,18,19]. On the left, the penicillin nucleus is shown in red; on the right, the cephalosporin nucleus is shown in green. For nucleus **A**, the stereochemistry is not defined because it is synthesized as a racemic 3,4-trans-β-lactam.

More recently, two other new classes of semisynthetic penicillins and cephalosporins, characterized by the presence of an additional β-lactam nucleus joined to the amino-nitrogen of 6-APA or 7-aminocephalosporanic acid (7-ACA), were reported in the literature (Figure 4) [16,17,18,19]. The additional 2-azetidinone nucleus **A** was characterized by the presence of two substituted phenyl rings at the C-4 and N-1 positions to favor hydrophobic interactions with the active site of β-lactamases and to improve the ability to inhibit them. 

All the synthesized 6-APA derivatives were tested in vitro against a variety of bacterial strains, representative of Gram-positive (*S. aureus*, *S. epidermidis*, *Bacillus sp.*) and Gram-negative bacteria (*E. coli*, *S. typhimurium*, *P. fluorescens*, and *P. aeruginosa*). The antimicrobial data indicated that all the compounds showed better activity against Gram-positive bacteria in comparison to Gram-negative bacteria, and, for *S. aureus* and *S. epidermidis*, the activity was higher than the reference drug. The compound with R = R_1_ = R_2_ = H was observed as the most active in the series. Additionally, in vitro cytotoxic studies against NIH-3T3 cell lines displayed no cytotoxic effects at concentrations higher than those proved to have antibiotic activity [16].

Next, after a computational study on the role played by two β-lactam moieties A and B (Figure 4) [17,18], a novel series of bis-β-lactam derivatives was synthesized, in which the 6-APA scaffold was replaced by the 7-ACA scaffold joint through an amide bond to the additional substituted β-lactam ring (Figure 4, right) [19]. Interestingly, the additional lactam ring was joined to the cephalosporin moiety by a chain of variable lengths (*n* = 1 to 7), assuming that the increasing of the distance between two moieties could increase the conformational flexibility and thus improve the interactions with the targets. All the synthesized compounds were tested against selected Gram-positive and Gram-negative bacteria strains and showed their effectiveness against Gram-positive bacteria. In particular, the compounds with an elongated carbon chain exhibited enhanced antibacterial activity compared to the reference drug. All the compounds displayed no in vitro cytotoxicity on tested MRC-5 and Calu-1 cell lines at the antimicrobial active doses.

**Figure 5:**
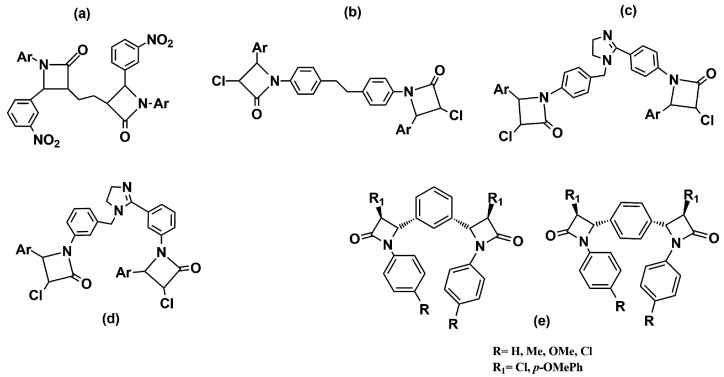
Chemical structures of bis-azetidinone connected by spacers of different types. Compounds (**a**–**d**) are reported in Refs. [20,21,22], compounds (**e**) in Ref. [23].

Other studies replaced the condensed bicyclic ring structures with monocyclic 2-azetidinone rings connected by spacers of various lengths and flexibility. The synthesized compounds were screened for their in vitro antibacterial activity against *B. subtilis*, *S. aureus*, *E. coli*, and *K. pneumoniae*. The results showed that all the compounds exhibited moderate to good activity, similar to that of the standard drugs. The compounds with a methoxy-, nitro- and amino-group on the phenyl ring at the C-4 position of the azetidinone ring were observed as the most active in the series (Figure 5a–d) [20,21,22]. 

Meenakshisundaram and coworkers [23] have reported related compounds with a phenyl ring as a linker between two azetidinones. All the derivatives shared, in their structures, two variously substituted 2-azetidinone nuclei linked at the C-3 position to the aromatic ring in the meta or para-relationship (Figure 5e). The compounds were subjected to an antimicrobial screening against nine strains of Gram-negative and Gram-positive bacteria. In general, all the compounds showed moderate activity, and usually, better activity against Gram-negative bacteria. In particular, among 1,4-positional isomers, the derivative with four chlorine atoms (R = R_1_ = Cl) in the structure exhibited good antibacterial activity against both Gram-negative bacteria (*P. mirabilis*, *P. vulgaris*, *M. morgana*, and *S. typhi*) and Gram-positive bacteria (*S. aureus*). Among the 1,3-positional isomers, the best antimicrobial activity was shown by the dimer with a chlorine atom on the C-4 position of 2-azetidinone nucleus (R_1_ = Cl), and a methyl substituent on the N-phenyl group (R = Me). Furthermore, the compound with a p-OMe phenyl ring at the C-4 position (R_1_ = p-OMePh) and a p-Me phenyl ring linked to the nitrogen N-1 of the β-lactam nucleus demonstrated an interestingly activity against a methicillin-resistant strain of *S. aureus* (MRSA), which was better than its corresponding monomer. 

**Figure 6:**
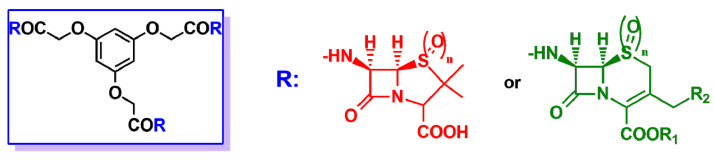
In the blue rectangle on the left, general structure of the β-lactam podand derivatives described in Refs. [24,25]. R may be either the 6-APA nucleus (red) or the 7-ACA nucleus (green), when n = 0, or the sulphone derivatives of 6-APA or 7-ACA when n = 2.

Another interesting structural class of multivalent β-lactam derivatives is β-lactam podands. Their structure is characterized by the presence of three nuclei of cephalosporin or penicillin arranged on a tricarboxylic acid acting as a central node (Figure 6). The antibacterial activity of the podands was tested on different strains of Gram-positive and Gram-negative bacteria, but only in some cases, significant results were found with an activity comparable to the antibiotic references [24,25]. 

**Figure 7:**
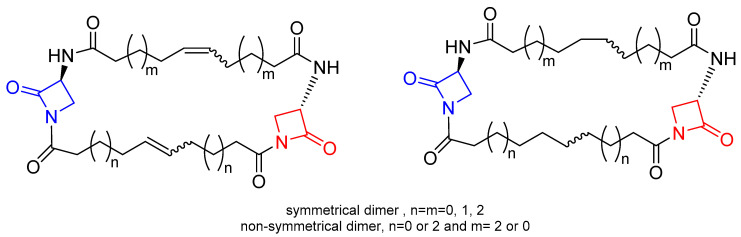
General structures of macrocyclic-embedded β-lactams reported in Ref. [26]. The azetidinone rings embedded in the macrocyclic scaffold are highlighted in red and blue.

Generally, the design of new potential β-lactam antibiotics has been aimed at improving the susceptibility of the β-lactam ring (acylating power) towards the nucleophilic attack of the target PBPs. An attractive approach is based on the synthesis of β-lactams embedded into macrocycles. The main idea of this approach is that the inclusion of the β-lactam moiety into a macrocyclic scaffold may facilitate the breaking of the N-C(O) bond of the 2-azetidinone ring, by increasing its conformational flexibility. The assumption is that the interaction of the β-lactam ring with the target enzymes involves a conformational reorganization during the formation of the acyl-enzyme intermediate. In this context, an extensive study was reported by Dive and coworkers [26]. A series of 1,3-bridged β-lactams embedded into macrocyclic scaffolds was synthesized (Figure 7) and the inhibitory activity against PBP2a (*S. aureus*) and PBP5fm (*E. faecium*), two representative PBPs of resistant bacteria, was evaluated. The results of the screening indicated that the series of the fully saturated compounds showed better activity against PBP2a than that of the unsaturated macrocycles, and the molecular shape and flexibility played a key role. In fact, for compounds of the same size (28 atoms), the non-symmetrical macrocycle was more active than the symmetrical one. In the series of the symmetrical dimers, the largest macrocycle was the most potent one, and the lower limit size to observe activity was the ring size of 24 atoms. However, none of the compounds showed inhibitory activity versus PBP5fm.

**Figure 8:**
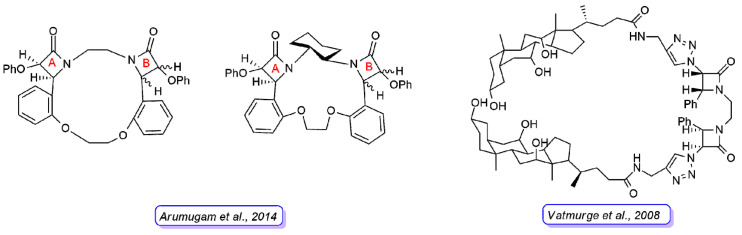
Representative structures of bis-β-lactam macrocycles reported in Refs. [27] (**left**) and [28] (**right**). For the compounds synthesized by Arumugam et al., the stereochemistry of the 2-azetidinone ring **A** is defined in all derivatives, whereas they differ in the stereochemistry of 2-azetidinone ring **B**.

Arumugam and coworkers [27] reported another series of bis-β-lactam grafted macrocycles (Figure 8). All the compounds were tested for in vitro antibacterial activity against four bacterial strains (*B. subtilis*, *S. aureus*, *P. aeruginosa*, and *K. pneumoniae*) and antifungal activity against *F. oxypsorium* and *M. phaseolina*. The results obtained indicated that the antibacterial activity was dose-dependent and the macrocycles with C_2_-symmetry (cis-anti-cis bis-β-lactam macrocycles) exhibited higher activity than *meso*-macrocycles (cis-syn-cis bis-β-lactam macrocycles).

Vatmurge and coworkers [28] have carried out the synthesis of some novel bile acid dimers containing two β-lactams as linkers to exploit the characteristic amphiphilicity of bile acids. The synthesized compounds were screened for their in vitro antibacterial activity against *S. aureus* and *E. coli* bacteria. The results indicated that none of the compounds exhibited comparable or better activity than the reference drugs against the *E. coli* strain. The compound shown in Figure 8 was the most active in the series against the *S. aureus* strain with an activity similar to the reference tetracycline and ampicillin.

### β-Lactam Nucleus on Calixarene Scaffolds

Calixarenes are a class of macrocycle compounds that are particularly interesting with “almost unlimited possibilities” [29]. Due to their intrinsic structural and physicochemical characteristics, they have been applied in different fields [30,31,32]. Particularly, calixarene derivatives have attracted much attention in recent years in medicinal chemistry due to their potential biological activity and their function as carriers or spatial organizers of various kinds of biologically active molecules [33,34,35]. The functionalization of the upper and lower rims allows for the possibility of introducing different moieties on the rigid calixarene scaffold with preorganized spatial relationships between the substituents. 

**Figure 9:**
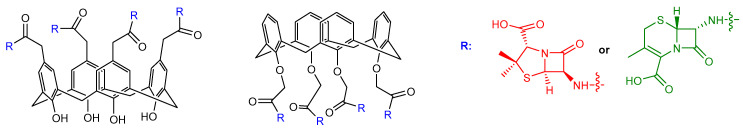
Calixarene derivatives bearing cephalosporin or penicillin nuclei at the upper or the lower rim reported in Refs. [36,37]. In calixpenams, R is the 6-APA nucleus (shown in red), in calixcephems, R is the 7-ACA nucleus (shown in green).

Examples of calixarene scaffolds functionalized with penicillin nuclei were reported by Pur and Dilmaghani. These calixarene-based penicillins represented a novel class of penicillin derivatives called calixpenams [36]. The synthesized compounds were functionalized at the lower or upper rim of the calixarene scaffold with four 6-APA (Figure 9). Their in vitro antibacterial activity was evaluated against different bacteria strains, showing that the calixarene derivatives were more potent than the monomeric counterparts against *S. pyogenes*, *S. agalactiae*, and *S. pneumoniae* bacteria strains. This result suggested a positive macrocyclic effect on the activity that provided a synergistic effect and a spatial preorganization of four 6-APA moieties. The most active compound was the derivative functionalized at the upper rim that the authors explained with a larger contact surface with the bacterial membrane due to the bigger size of the upper rim compared to the lower rim. The same group synthesized new calixarene derivatives with cephalosporin nuclei linked to their scaffold (Figure 9) and called them calixcephems [37]. The calixcephems displayed enhanced antibacterial activity, with minimum inhibitory concentration (MIC) values from 5 to 10-fold lower in comparison to the corresponding single cephem and parent calixpenam. Notably, the compound with all cephem moieties at the lower rim was more effective against MRSA compared to the corresponding calixpenam.

## 3. β-Lactam Antibiotics Bearing Additional Pharmacophores

### 3.1. Hybrid Antibiotics with a β-Lactam Moiety Associated with Another Antibiotic Moiety

A strategy to overcome the possibility of a failure during antimicrobial therapy is to administer a combination of different drugs, to expand the spectrum of action and/or to counteract the insurgence of resistant bacterial strains. Attempts to combine antimicrobial drugs arose since the 1960s at the end of the “golden era” and the start of the “lean years” in which medicinal chemistry reached the lowest point of new antibiotic discovery and development, together with a parallel increase in antibiotic resistance [11]. This situation pushed clinicians to combine two or more molecules with different biological targets. In this way, many associations between different molecules became standard when they were found to be more effective than the individual compounds. This is the case of the well-known association of sulfonamides with trimethoprim [38]. However, examples of formulations of fixed-dose antibiotics combinations are rare, and most combined therapies are empirically applied in clinical practice [39]. While most of these treatments were found to have some success, at least against infections caused by Gram-positive bacteria, their benefit for delaying resistance development has been questioned [40]. Nonetheless, exploring new ways to combine different drugs would probably counteract the increasing spreading of resistance and alleviate the difficulty of discovering innovative molecules.

Some challenges in the co-administration of two different antibiotics could be overcome by synthetically linking individually bioactive components into a single molecule. This is the rationale for the development of the so-called “hybrid antibiotics”, i.e., molecules composed of two or more pharmacophores with different antimicrobial properties. These molecules appear to have a promising future since they usually behave as novel entities with activity superior to the simple sum of the activity of the constituent agents [41]. For this reason, they are expected to expand the current portfolio of antimicrobial weapons [42]. In this section, we will focus on those hybrid antibiotics containing at least one β-lactam moiety. In several cases, the association of β-lactam antibiotics with other pharmacophoric moieties was aimed at the release of the other pharmacophore once the β-lactam ring is cleaved by β-lactamases, whereas in other cases, hybrid antibiotics behave as a truly dually active molecule [40].

The first example of this type of compounds was probably a cephalosporin derivative synthesized at Glaxo laboratories in 1976, bearing, in position 3, the antiseptic pyrithione, also known as omadine (a substituent that possesses an antibacterial activity per se). This compound showed activity against β-lactamase-bearing *E. coli* strains, causing bacterial lysis followed by a prolonged bacteriostatic effect [43,44]. Interestingly, more than 40 years later, pyrithione-containing cephalosporins were found to be effective against strains of *M. tuberculosis* under both replicating and non-replicating conditions, supporting the idea that two different mechanisms of action can kill mycobacteria in different metabolic states [45]. 

Other examples were published in which a cephalosporin was coupled to an antibiotic dipeptide [46]. More recently, Mobashery and coworkers designed dual antibiotics formed by β-lactams and aminoglycosides, to reduce the toxicity of the latter pharmacophore and to enhance its penetration into bacteria. Three conjugates prepared by this research group, in which the two pharmacophores were connected by a carbamate spacer, were biologically tested and showed lower toxicity than the aminoglycosidic compound alone [47]. Other dual antimicrobial compounds, including nitrofurantoin, clioquinol, cycloserine, and oxolinic acid, linked to the penem β-lactam ring were obtained by Farmitalia Carlo Erba [48]. Researchers in Bristol-Myers Squibb developed β-chloro-L-alanine-containing carbapenems [49]. In this case, the release of the β-chloroalanine moiety occurs without the cleavage of the β-lactam ring, revealing that those molecules are actually bifunctional compounds [50]. Another association was attempted with phloroglucides, but the ester derivative linked to position 3 was less active against several bacterial strains, with respect to phloroglucides alone. On the contrary, the derivative linked to position 7 of the cephalosporanic nucleus was more resistant to β-lactamase, probably because of the steric hindrance of the phloroglucide moiety [51]. Furthermore, the cephalosporin scaffold was associated with triclosan, acting as an enoyl-ACP-reductase inhibitor, but the activity of triclosan was dependent on the cleavage of the β-lactam ring [52,53]. 

An extensively studied combination of hybrid antibiotics was the one linking β-lactam derivatives with antibiotics belonging to the quinolone/fluoroquinolone family. This combination seems to be the ideal one because the resulting molecules have a broad spectrum of activity. Indeed, quinolone and fluoroquinolone derivatives act against several bacterial targets, such as DNA gyrase, topoisomerase II, and IV, which do not interfere with the targets of β-lactam antibiotics. Moreover, quinolone and fluoroquinolone derivatives are highly active against Gram-negative bacteria, whereas β-lactam antibiotics are usually more focused against Gram-positive bacteria. Thus, the hybrid compound can have a broader spectrum of action with respect to each precursor. Additionally, both families of compounds allow for the introduction of several chemical modifications without losing antibacterial activity. Finally, the fabrication of these hybrid antibiotics enhances the solubility of the fluoroquinolone moiety [54]. Roche was particularly involved in the synthesis and evaluation of many hybrid antibiotics in which cephalosporin moieties were bound to quinolones/fluoroquinolones [55]. Some of these hybrid compounds showed in vitro antibacterial activity that was compatible with a two-stage activity: first, the β-lactam moiety exerts its antimicrobial action, and once it is disrupted by β-lactamases, the quinolone moiety can start its activity. However, cephalosporin 3′-quinolone, bound together by carbamates and tertiary amine linkages, appeared to act as intact molecules with complementary spectra of action. Derivatives containing a tertiary amino group showed a broad antibacterial activity, whereas derivatives with a spacer containing a quaternary amino group were found to be highly stable in solution, but with lower antibacterial activity [56,57,58,59,60]. The design of a dual cephalosporine-quinolone compound linked by a tetrazole-containing spacer was attempted to allow the release of the fluoroquinolone, following cleavage of the β-lactam ring. The resulting compound was found to have enhanced stability in solution, but moderate activity in vitro, probably because the planned hydrolysis of the tetrazole-containing spacer is only effective at high pH values [61]. 

**Figure 10:**
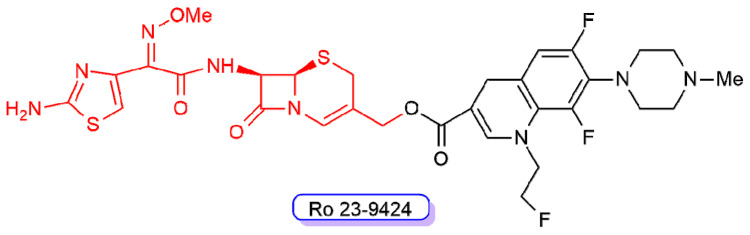
Chemical structure of compound RO-239429. The third-generation cephalosporin scaffold is highlighted in red, whereas the fluoroquinolone scaffold is shown in black.

One of these cephalosporin-quinolone derivatives, Ro 23-9424 (Figure 10), was obtained by combining the third-generation cephalosporin desacetylcefotaxime with the third-generation fluoroquinolone fleroxacin. Ro 23-9424 was found to have promising properties and reached clinical trials [62,63,64,65,66,67]. However, no further progress of this molecule into clinics has been reported to date.

Probably the most recent example of a combination of a cephalosporin moiety and a quinolone is the cephalosporin-fluoroquinolone hybrid molecule developed by Evans and coworkers [68]. Their compound has been designed to be selectively activated in bacteria expressing β-lactamase. To achieve this goal, the researchers optimized the β-lactam moiety in order to reduce the activity of the intact molecule and enhance the release of quinolone moiety. In this way, it was possible to achieve a therapeutic activity minimizing selection for resistant strains and with no damage to microbiota. This indicates that these compounds can probably be further exploited for clinical applications. 

The same principle was applied to penems and carbapenems, by synthesizing derivatives in which the quinolone moiety was joined at the C-2′ position of the β-lactam scaffold through an ester or carbamate linkage showing potent and broad antibacterial activity reflecting the contribution of both pharmacophores [48,69,70,71]. 

**Figure 11:**
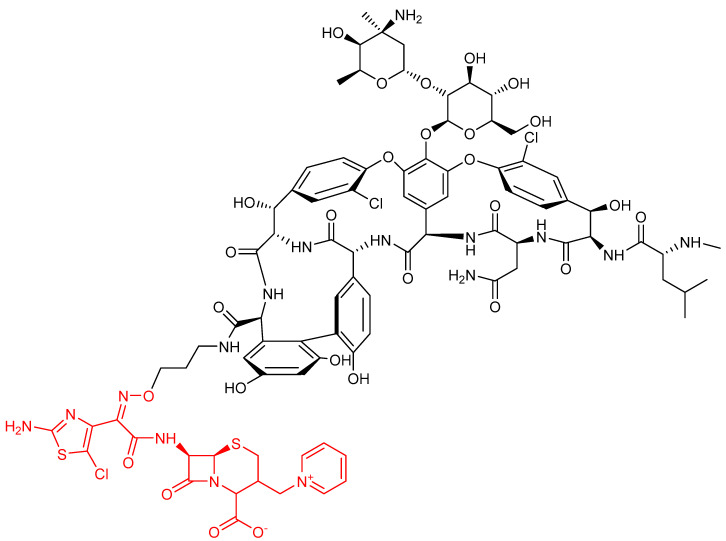
Chemical structure of compound TD-1792. The cephalosporin moiety is highlighted in red.

Among other hybrid antibiotics bearing a β-lactam moiety, another promising association seems to be the one with glycopeptides, in particular vancomycin. Indeed, both pharmacophores block the cell wall synthesis in bacteria. The proximity of their targets induces thinking that this chimeric structure can allow the 2-stage block of this process, thus greatly enhancing the antibacterial activity of each pharmacophore. Several derivatives of this family were studied by combining different options to couple the two molecules [72], and the most promising one, developed by Theravance BioPharma and R-Pharm, is the one identified as TD-1792 (Figure 11). This compound has a very complex structure in which a third-generation cephalosporin (patented as THRX-169797) is attached to the vancomycin core through a direct amide linkage at the C-terminus of vancomycin.

This compound, together with others, synthesized with the same strategy, was in vitro screened against several Gram-positive bacterial strains, including MRSA, and showed excellent potency (MIC of 0.05 μg/mL), whereas the individual antibiotics were about 30-fold less potent. A structure-activity relationship analysis revealed that the relative spatial orientation of the two components was not critical to increase the potency. Thereby, the authors inferred that the simultaneous binding of this hybrid antibiotic to two cellular targets is unlikely, and a possible mechanism of action is the interaction with the transpeptidase site of PBP2 and the D-Ala-D-Ala dipeptide involved in cross-links to stabilize the peptidoglycan. This compound was also tested in an in vivo model of MRSA infection, confirming its higher effectiveness with respect to vancomycin alone [73]. Its efficacy was further proved against vancomycin-resistant clinical isolates of *Staphylococcus* spp. (MIC_90_ up to 8-fold lower than that of other well-known antibiotics, even in the presence of human serum) [74] and against several other bacteria, including clinical isolates of methicillin-sensitive strains of *S. aureus* (MSSA) and MRSA [75]. Notably, in this last study, the activity of TD-1792 was compared to that of the cephalosporin moiety alone, and the latter resulted in less activity, suggesting a synergistic mechanism of action between the two components of this hybrid antibiotic. Moreover, the coexistence of resistance to methicillin and vancomycin in these strains did not affect the antibiotic activity of this compound. The activity against anaerobic Gram-positive bacteria was also proved, whereas this molecule shows poor activity against most Gram-negative anaerobic bacteria [76]. Pharmacokinetic and pharmacodynamics studies in a murine model confirmed potent antibacterial efficacy, in vivo, against all microorganisms tested at doses of 10 mg/kg and lower, and the potency was unaffected by changing the dosing interval [77]. A further randomized, double-blind, active-control, phase II trial in patients with complicated skin infections caused by Gram-positive organisms, including MRSA, showed that TD-1792 and vancomycin had similar cure rates, but TD-1792 achieved the highest cure rate in patients infected with MRSA. Moreover, adverse effects were mild and comparable to those caused by vancomycin alone [78]. TD-1792 is currently in phase III trials for skin and soft tissue infection in Russia and Georgia (update 31 January 2020, from https://adisinsight.springer.com/drugs/800020586). 

Some examples of dual compounds, linking a β-lactam moiety with an antitumor agent together, were also obtained. In most cases, the main goal of these hybrid compounds was to decrease the toxicity of the chemotherapeutic compound by employing an approach involving a monoclonal antibody targeting tumor-associated antigens, conjugated to an enzyme for the selective activation of a prodrug only able to release the chemotherapeutic compound in the site of tumor [79]. Few hybrid β-lactam antibiotics bound to cytotoxic agents such as the mitozolomide or temozolomide rings were synthesized to exploit the cytotoxicity of the latter compound to improve the antibacterial activity, but the results were not particularly encouraging [80].

### 3.2. Hybrid Antibiotics with a β-Lactam Moiety Associated with Other Non-Antibiotics Moieties

The most important mechanism in developing resistance towards β-lactam antibiotics is mainly linked to the spreading of β-lactamase-mediated resistance [81]. A possible way to counteract this mechanism of resistance is to co-administer β-lactam antibiotics together with other compounds that are able to block these enzymes. This is the rationale of the well-known association between β-lactam antibiotics and β-lactamase inhibitors, which are molecules that show weak antibiotic activity but can bind to these enzymes and act as suicide inhibitors, thus encompassing bacterial resistance [82].

Another well-known mechanism of β-lactam resistance developed by many bacteria affects the ability of the antibiotics to encompass the bacterial cell wall [81]. Moreover, many β-lactam antibiotics show limited activities against Gram-negative bacteria because the structure of the cell wall of these microorganisms is much more complex than that of Gram-positive bacteria, and is much more efficient in inhibiting the permeation of external substances. 

Many different strategies have been adopted to enhance the permeability of β-lactam antibiotics across the bacterial cell wall [83], and some of them involve the creation of dual compounds in which one moiety improves the passage through the cell wall of the β-lactam derivative. For example, penicillins and cephalosporins were combined with aminoquinoline to obtain dual-action agents in which the pharmacophoric group of aminoquinoline served as an inhibitor of membrane efflux pumps. Only the cephalosporin derivative, however, showed high antibacterial activity against Gram-positive strains, but the activity against Gram-negative bacteria was still moderate [54].

Some promising approaches involve the use of siderophore-antibiotic conjugates, a so-called “Trojan-horse approach” to enter the periplasmic space, avoiding the degradation by several β-lactamases. Siderophores are small (150–2000 Da) molecules produced by almost all bacterial species and involved in iron uptake from the environment. More than 500 different molecules have been identified; they are characterized by a large structural diversity, but common functional groups to coordinate iron, in particular catechols, hydroxamates, phenolates, carboxylates, and their combinations [84]. These molecules chelate extracellular iron ions with a very high affinity, and promote their passage across the cell wall by binding specific receptors that internalize the complex via active transport. This uptake system is therefore a very important way to overcome the barrier represented by the cell wall of Gram-negative bacteria. Natural antibiotics called “sideromycins” formed by a siderophore moiety and a toxic group were found to exert their activity thanks to the fact that they can penetrate bacterial cells using this system [85]. Therefore, the conjugation of known antibiotics with siderophores mimics these natural compounds and represents an opportunity to increase the delivery to their molecular targets [86]. 

Given that the target of β-lactam antibiotics is located in the periplasmic space of bacteria, and that this class of antibiotics tolerates well extensive substitutions (indeed, the antibiotic activity does not require a release of the antibiotic from the conjugate), this strategy has been extensively used, especially for this class of antimicrobial compounds [87]. The siderophore group binds the iron, and the iron-antibiotic complex crosses the outer membrane and accumulates a high concentration into the periplasmic space, where iron dissociates, allowing the antibiotic pharmacophore to bind to PBPs. 

The first examples of the conjugation of penicillins and cephalosporins with siderophores date back to the 1980s [88,89,90,91,92,93,94,95,96,97] and it was soon probed that their enhanced activity against Gram-negative bacteria was due to the outer membrane permeation via iron-regulated transporters [98]. In some cases, natural siderophores, such as enterobactin, salmycins, agrobactin, parabactin and fimsbactin, were exploited, either unmodified or slightly modified, to create conjugates with β-lactam antibiotics (extensively reviewed in References [99,100]). Many other examples of a synthetic siderophore, coupled to β-lactam antibiotics, have also been published (extensively reviewed in Reference [87]). Among the possible synthetic siderophores, catechol- and hydroxypyridone-conjugates were preferred for penicillins and cephalosporin-derivatives, whereas hydroxamate conjugates were less exploited [101].

Monobactams and monocarbams (monocyclic β-lactams with a sulfonylamino carbonyl group bound to N-1) were mainly conjugated to hydroxypyridone and gained enhanced potency against Gram-negative bacteria, but no inhibition of β-lactamases [102,103,104,105,106]. The linker components of the conjugate were also extensively studied and many different cleavable and non-cleavable functional groups have been tested [99], including even β-lactam moieties [107]. As anticipated, usually β-lactam antibiotics do not require cleavage for being activated, whereas cleavable linkers are needed in the case of conjugates with other antibiotics, especially those with cytoplasmic targets [108,109].

Despite the efforts worldwide to identify antibiotic-siderophore-conjugates suitable for therapeutic use, only a few molecules have effectively entered clinical trials. For several compounds, the reason was simply due to the lack of interest of industries in developing these compounds [87], but in other cases, the reason was due to the variable in vivo efficacy, often not predictable by in vitro tests. Most failures were probably due to a combination of causes, such as the low affinity of synthetic siderophores for the iron, the lack of recognition of these compounds by bacterial receptors, the inactivation of antibiotics due to the necessary modifications to bind the two moieties together, the use of non-optimal linkers, and acquired resistance [110]. Surprisingly, in some cases, the conjugation of antibiotics with siderophores was found to promote bacterial growth, because the iron uptake was prevalent over the antimicrobial activity [111,112]. In many cases, the acquired resistance by the bacteria was due to modifications in membrane transporters [113,114,115].

Among those few molecules that reached clinical trials, it is worth mentioning the siderophore-monosulfactam BAL30072, developed by Basilea Pharmaceutica, which showed potent activity against several Gram-negative bacteria and was a poor substrate for many β-lactamases [116,117,118,119,120]. This promising molecule entered phase I clinical trials, but its development was subsequently stopped [86]. Another compound that reached phase I clinical trials was cefetecol, a catechol-conjugated cephalosporin with potent in vitro activity, but poor in vivo efficacy [86]. This failure has been attributed to the methylation of catechol moiety by mammalian catechol-O-methyltransferase, as this modification impairs the recognition of the former siderophore by its bacterial transporter [121].

**Figure 12:**
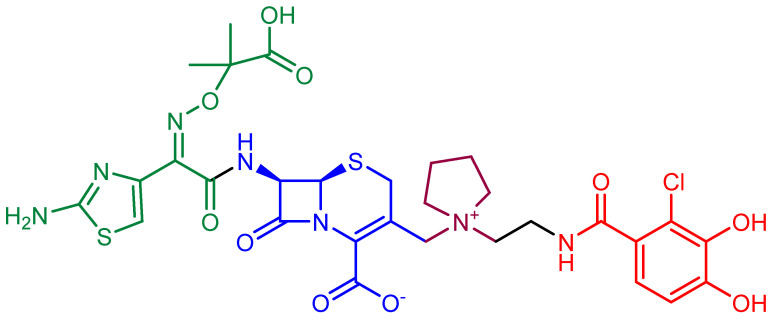
Chemical structure of cefiderocol. The cephalosporin core is in blue, the green part is the sidechain inspired by ceftazidime, the purple part is the sidechain inspired by cefepime, and the red moiety is the siderophore.

The most successful example of an antibiotic-siderophore conjugate is cefiderocol [122] (Figure 12), a cephalosporin derivative developed in 2014 by Shionogi & Co. The cephalosporin core is inspired by those of ceftazidime (a third-generation cephalosporin) and of cefepime (a fourth-generation cephalosporin), both active against Gram-negative bacteria. A side chain containing both the aminoacyl group and the aminothiazole ring attached to the alpha carbon increases the microbiological spectrum of activity. The dimethylacetic acid moiety attached to the imino group, similar to that of ceftazidime, increases the activity against *P. aeruginosa*. The addition of the positively charged quaternary ammonium group, as in cefepime, promotes the penetration of the molecule across the outer membrane of Gram-negative bacteria. Finally, the catechol extending from this quaternary ammonium group is the siderophore moiety. 

Cefiderocol has proved to be effective in vitro against a high number of Gram-negative species, including *E. coli*, *K. pneumoniae*, *S. marcescens*, *C. freundii*, *A. baumannii*, *P. aeruginosa*, *S. maltophilia*, *B. cepacia*, and many other multidrug-resistant bacteria [123,124,125,126,127,128,129,130,131]. Moreover, this antibiotic appears to be resistant to the hydrolysis of most β-lactamases [132,133,134] and its uptake is mildly influenced by the down-regulation of porins [133]. Many clinical trials and case reports (reviewed in Reference [122]) supported the safety and efficacy of this antibiotic against a broad spectrum of infections caused by Gram-negative bacteria. Currently, further information about phase III clinical trials is waiting to be published, in order to figure out a possible role of this compound in the therapy.

### 3.3. Conjugates with Other Non-Antibiotic Activities

The family of tetramic acids is a large group of compounds bearing a common structural unit: a pyrrolidine-2,4-dione ring system or its corresponding enolic form, embedded in simple heterocycles or complex structures with long chains or fused polycyclic skeletons. Frequently, position 3 of the tetramic acid ring is substituted by an acyl moiety, and position 5 is often substituted by amino acid derivatives [135]. The first derivative isolated was L-tenuazonic acid, in the fungus *A. alternate* [136], but in the last decades, many members of this family have been isolated from diverse environments [135]. Tetramic acid derivatives show a wide variety of biological activities, including antibiotic, antiviral, mycotoxic, cytotoxic, and phytotoxic properties, with diverse modes of action [137], but although they have been known for a long time, the study of their possible applications in medicine is much more recent [138]. 

3-acyltetramic acid derivatives show the ability to coordinate many different metal ions, including Ca^2+^, Mg^2+^, Cu^2+^, Fe^3+^, and Ni^2+^, and this chelation is requested to exploit their biological activity [139,140]. They were found to act as siderophores in several bacteria [141]; thus, some researchers tried to exploit this activity by creating tetramic acid-β-lactam derivatives. The first attempt to obtain such derivatives was made by Cherian and coworkers, which attached the 3-acyltetramic acid moiety to the amine terminal of ampicillin in order to increase its permeability across the membranes and to create molecules similar to ureidopenicillin, known for their broad-spectrum activity [142]. The resulting compounds showed moderate activity against some Gram-positive bacteria, but promising activity against MSSA, *Streptococcus* spp. and *Bacillus* spp., and some activity against Gram-negative bacteria, in particular against *P. aeruginosa*. Structure-activity relationship studies of these compounds suggested that these hybrids could bind to PBPs with suboptimal affinity, and they were found to be substrates for both β-lactamases and efflux pumps. Surprisingly, it turned out that they did not act as siderophores, but they probably exploit other mechanisms for their uptake [137]. Further studies made by the same research group allowed to obtain new β-lactam-tetramic acid derivatives with improved antibacterial activity. In particular, several cefaclor and cephalexin-tetramic acid derivatives were found to inhibit the growth of MRSA at low concentrations, with potency higher than that of cefaclor and cephalexin alone. Generally, these compounds showed no activity against Gram-negative bacteria. On the contrary, cephamycin-tetramic acid analogs were inactive against Gram-positive bacteria, but showed good activity against Gram-negative bacteria [143].

## 4. Conclusions

β-lactam antibiotics are among the oldest classes of antimicrobial compounds known, but despite their venerable age, they are still used diffusely in the clinics because of their high specificity, potency, and safety coupled to their low costs of production. A feature that has made them able to continuously renew themselves over the years is their chemical plasticity, which has allowed them to generate many different molecules, starting from the common β-lactam scaffold. 

Among the thousands of different modifications, a peculiar niche, perhaps less explored than others, is made of molecules coupling two β-lactam rings, either directly or using different linkers, to obtain a bis-azetidinone molecule. Some research groups, including us, have attempted this strategy with the focus on developing antibiotics mimicking the combination between an antibiotic and a β-lactamase inhibitor. At present, no molecule of this class has attained the clinics, but in our opinion, there is room for improving them in terms of the spectrum of action and the ability to defeat some of the most dangerous strains of multi-drug resistant bacteria.

Other classes of bifunctional compounds involving β-lactam derivatives are those in which another antimicrobial function is linked to the β-lactam moiety, to create a permanent association between two different antibiotics. One promising example of such a bifunctional compound is TD-1792, which is currently in phase III clinical trials.

Finally, a further class of bifunctional compounds is the one in which the spectrum of action of β-lactam compounds has been broadened by exploiting some peculiar bacterial transporters, thus allowing these molecules to overcome the cell wall, in particular in Gram-negative bacteria. Some examples of this group include siderophore and tetramic acid-conjugates, and one convincing molecule, cefiderocol, is completing phase III clinical trials.

However, it cannot be ignored that most efforts in this area seem to have produced few results. Many are the possible causes of this apparent disproportion between efforts and results. First, all these molecules are much larger than the average size of drugs; this may induce researchers to discard them in the early stages of the drug development process. However, it is worth noting that several compounds in clinics violate Lipinski’s rules, so perhaps more attention would be needed before discarding potentially valid compounds, only for their magnitude, before evaluating their in vivo effectiveness. Second, it is true that the synthesis of these compounds is often complex, especially when the different classes of antibiotics to be coupled have different chemical properties. However, we think it is worthwhile to study new chemical strategies to make these compounds because this could also have positive effects on the synthesis of monofunctional compounds with different substituents, thus significantly expanding the libraries of the compounds that are available. Finally, it appears that the further development of these classes of antibiotics was also conditioned by the strategic plans developed by the pharmaceutical industries, which, faced with the high risks of producing antibiotics that are rapidly overcome by antibacterial resistance and the high costs of managing the development of these compounds compared to the low profits obtainable from them, have often decided to stop the production of antibiotics [144]. However, the COVID-19 pandemic we are experiencing teaches us that we must not let our guard down in the face of the possible development of lethal microorganisms that can have dramatic consequences for humanity. Therefore, we hope that the pressing demand for novel antibiotics will prompt pharmaceutical industries to rethink these decisions. We also hope that a new model of cooperation among pharma, academic research, governments, and other subjects (e.g., charitable foundations) will bring the search for new antibiotics back into the foreground needs of pharmaceutical research. 

## Data Availability

No new data were created or analyzed in this study. Data sharing is not applicable to this article.

## Abbreviations

PBP	Penicillin-Binding Protein
6-APA	6-aminopenicillanic acid
7-ACA	7-aminocephalosporanic acid
MRSA	Methicillin-resistant strain of *S. aureus*
MSSA	Methicillin-sensitive strain of *S. aureus*
MIC	Minimum inhibitory concentration
